# Stigma among ebola disease survivors in Mubende and Kassanda districts, Central Uganda, 2022

**DOI:** 10.1371/journal.pgph.0003272

**Published:** 2024-12-16

**Authors:** Marie Gorreti Zalwango, Sarah Paige, Richard Migisha, Brenda Nakafeero Simbwa, Edirisa Junior Nsubuga, Alice Asio, Zainah Kabami, Jane Frances Zalwango, Peter Chris Kawungezi, Mercy Wendy Wanyana, Patrick King, Hellen Nelly Naiga, Brian Agaba, Robert Zavuga, Giulia Earle-Richardson, Benon Kwesiga, Lilian Bulage, Daniel Kadobera, Alex Riolexus Ario, Julie R. Harris

**Affiliations:** 1 Uganda Public Health Fellowship Program-Uganda National Institute of Public Health, Kampala, Uganda; 2 United States Agency for International Development, Kampala, Uganda; 3 National Center for Emerging Zoonotic and Infectious Diseases, US Centers for Disease Control and Prevention, Atlanta, Georgia, United States of America; 4 Division of Global Health Protection, US Centers for Disease Control and Prevention, Kampala, Uganda; Brigham and Women–s Hospital, UNITED STATES OF AMERICA

## Abstract

Ebola disease survivors often experience stigma in multiple forms, including felt (perceived) stigma, enacted (action-based) stigma, and institutional stigma. On September 20, 2022, Uganda declared a Sudan Virus Disease (species *orthoebolavirus sudanense*) outbreak after a patient with confirmed Sudan virus (SUDV) infection was identified in Mubende District. The outbreak led to 142 confirmed and 22 probable cases over the next two months. We examined the types of stigma experienced by survivors and their household members and its effect on their well-being. We conducted a qualitative study during January 2023 in Mubende and Kassanda Districts. We conducted in-depth and key informant interviews with ten SUDV disease survivors, ten household members of SUDV disease survivors, and ten key informants (district officials and health workers in the affected communities). Interviews were recorded, translated, transcribed, and analyzed thematically. Survivors reported experiencing isolation and rejection by community members and loss of work. They reported being denied purchases at shops or having their money collected in a basket and disinfected (enacted stigma), which led to self-isolation (felt stigma). Educational institutions denied admission to some students from affected homes, while parents of children in some affected families stopped sending children to school due to verbal abuse from students and teachers (structural stigma). Prolonged SUDV disease symptoms and additional attention to survivors from responders (including home visits by health workers, public distribution of support items, and conspicuous transport from home to the survivor’s clinic) were perceived as aggravating both felt and enacted stigma. Even after the outbreak had been declared over, survivors felt that they were still considered a threat to the community. Survivors experienced mainly enacted stigma which was aggravated by the outbreak response and control activities such as additional attention to survivors from responders. Strengthening community engagement to counteract stigma, rethinking response activities that aggravate stigma, integrated response interventions by partners, private distribution of support items, and increasing awareness and sensitization could reduce stigma among the Ebola disease survivors in future responses.

## Introduction

Ebola disease is a severe and frequently lethal disease caused by infection with orthoebolaviruses. Ebola disease outbreaks typically start following exposure of a human to the body fluids of an infected bat or primate, followed by human-to-human transmission through contact with infected bodily fluids or contaminated fomites [1]. There are six species of *orthoebolavirus*: species *orthoebolavirus bundibugyoense*, species *orthoebolavirus zairense*, species *orthoebolavirus sudanense*, species *Orthoebolavirus restonense*, species *Orthoebolavirus bombaliense*, and species *orthoebolavirus taiense*. Species *orthoebolavirus bundibugyoense*, species *orthoebolavirus zairense* and species *orthoebolavirus sudanense* have caused epidemics in Africa, with case-fatality rates ranging from 25–90% in the absence of treatment [2–4].

Several ebola disease outbreaks have been registered in Africa in the last three decades [5]. Uganda has registered five ebola disease outbreaks since 2000 caused by Species *orthoebolavirus bundibugyoense* and species *orthoebolavirus sudanense* [6, 7]. On September 20, 2022, Uganda declared the sixth ebola disease outbreak in the country, caused by Sudan virus (SUDV). Sudan virus disease (SVD) has been the most frequent ebola disease in Uganda [8]. The outbreak which affected nine districts with Mubende and Kassanda districts having the vast majority of cases led to 142 confirmed cases, 22 probable cases and 87 survivors [9].

Survivors of all forms of ebola disease including Sudan Virus disease often experience stigma which can result into mental health challenges [10–14]. This stigma among ebola disease survivors is primarily associated with fear of ongoing contagion, and has led to evictions, intimate partnership dissolution, termination of employment, abandonment, and physical violence [15]. Stigma may take three forms: enacted stigma where survivors are discriminated against by community members, felt stigma where the stigmatized persons endorse the negative views from others and institutional stigma where institutions such as medical, educational, and employment institutions perpetuate the stigma [16]. Understanding the distinction of the various forms of stigma experienced is critical in identifying the various strategies required for control.

Stigma can affect the willingness of affected persons to seek care and the community’s acceptance of prevention and case management packages [6]. As a result, addressing disease-associated stigma starting early in an epidemic through comprehensive medical and psychosocial support and community interventions can be critical. Support provided to survivors after discharge has been linked to better coping, accelerated recovery, and faster restoration of their sense of dignity [17]. Furthermore, community efforts, such as training survivors as peer health promoters to support disease prevention and response efforts, may also help in the psychosocial recovery of individuals and in their de-stigmatization within communities [18].

Anticipating stigmatization of survivors after the outbreak, the Uganda Ministry of Health and its development and implementing partners put in place multiple interventions to reduce stigma and quicken reintegration of survivors in the communities. Starting in November 2022, the Ministry of Health, supported by USAID, CDC, and other partners, established a national Ebola survivors program to support the 87 survivors of the 2022 Uganda outbreak [19]. Support activities included community dialogue meetings during which survivors, their families, and communities received information about SUDV and how to help the survivors continue to recover. In addition, response staff traveled to communities to conduct health education using loudspeakers. Survivors were provided with 400,000 Uganda shillings (approximately $100 USD) each from the Bangladesh Rural Advancement Committee (BRAC) Uganda to help compensate for time lost at work. They further received in-kind support such as mattresses, clothes, and food from UNICEF and Save the Children. These supplies were issued publicly at the subcounty headquarters during November 2022 to January 2023 [20]. The survivors’ clinic provided a branded van to collect survivors from the community and bring them in for biweekly physical checkups and psychosocial support; survivors’ clinic staff and other health partners also provided psychosocial support to survivors and their family members during visits to the affected communities. Despite these efforts, stigma may continue to negatively affect ebola disease survivors. To better understand how to promote the well-being of survivors in Uganda, we examined the stigma experienced, aggravating factors, effects of stigma experienced by ebola disease survivors and their household members to inform stigma control measures for improved epidemic response and survivor support in future outbreaks. We further explored opportunities for reducing stigma through key informant interviews conducted among local government leaders on stigma reduction recommendations.

## Methods

### Study design and setting

We conducted a qualitative study to explore the perceptions and experiences of SVD survivors and their household members using in-depth interviews of survivors, household members, and local government officials with knowledge of the issue (“key informants”). We focused on Mubende and Kassanda districts since these were the most affected areas and had a large number of SVD survivors. Neither of these districts had experienced an ebola disease outbreak before.

### Study population

Participants for this study were SVD survivors in the Mubende and Kassanda districts and members of the same household as the survivors aged 18 years or above. However, an exception was made to include emancipated minors (children above 16 years living independently). In these two regions, there are a total of 63 known survivors [40 (63%) men, 23 (37%) women and 9 (14%) children <18 years]. Of the 63 survivors, 36 (57%) were from Mubende District. Children below 18 years (with the exception of emancipated minors) and individuals whose physical and psychological health limited their ability to provide information through the interview process were excluded from this study. We identified survivors using a discharge list from the Ebola treatment unit (ETU) and located them in the communities with the help of community health workers (CHW). All participants provided written informed consent to participate.

We actively searched for survivors and their household members from each district with the help of community health workers and a guiding list of discharged patients obtained from the Ebola treatment unit. Engagement for in-depth interviews based on their availability at home and wiliness to participate in the study. Only one survivor or household member was recruited per household for in-depth interviews to increase the variability of findings. We also engaged local government leaders from each district as key informants on how stigma could be reduced for improved wellbeing of the SVD survivors. These included health workers attached to health facilities in SVD-affected communities, the District Health Officer (DHO), Resident District Commissioner (RDC), and the District Surveillance focal person (DSFP). Saturation of information was reached with 20 in-depth interviews (10 survivors and 10 household members) and 10 key informant interviews.

### Data collection

An interview guide with open-ended questions and discussion prompts related to survivor feelings and experiences was used to conduct in-depth interviews in the local language (Luganda) with the SVD survivors and their household members. For survivors and their household members, we collected data on demographic characteristics, number of SVD patients in household, and SVD deaths in the household. We further explored: experiences of stigma, possible stigma instigating factors or actions, how stigma affected their lives, possible suggestions for the control of stigma, and any additional support required. For Key informant interviews, we explored community perceptions and actions towards survivors and their household members, health concerns of survivors, and recommendations for improvement. Information from the interviews was recorded in electronic form using audio digital recorders. Data were collected from participants in both districts concurrently until saturation was attained [21].

### Data analysis

Recordings were translated to English and transcribed at the end of each data collection day and stored by the principal investigator in a password protected computer. Participants were given an identification number based on the type of interview and order in which they were interviewed.

After data collection, transcripts were reviewed by the interviewers, coded and analyzed thematically using the CDC Excel Tool for Thematic Analysis V2.0 (10.18.22) (“Excel Tool”) to bring out the story of lived experiences and recommendations. The same coding scheme was used for all the 30 transcripts at the same time. The initial coding was done by the main operator and later verified by 2 other researchers to ensure that all relevant statements to the objectives under study were accommodated in the existing codes. We reviewed the common codes and created statements about stigma that one or more common codes suggested. Once all of the texts were fully coded in the CDC tool, researchers created simple tables showing the frequency distributions of the codings, with the most frequently used codes at the top, and the least frequently used codes at the bottom. This provided a first look at common codes to consider for development into themes. This also allowed researchers to doublecheck coding and decide if any revisions to the coding scheme were needed. Using the coded text, we developed themes around stigma among SVD survivors.

### Ethical considerations

Before starting the project, a non-research determination form was submitted to the US Centers for Disease Control and Prevention (CDC) as a requirement. The Office of the Associate Director for Science at the CDC determined that the project did not involve human subjects research. This determination was made because the project aimed to address a public health problem and had the primary intent of public health practice. Further administrative approval to conduct this study was obtained from Mubende and Kassanda District offices, Mubende regional referral hospital case management team, the National Institute of Public Health, and Uganda Ministry of Health. Before data collection, written informed consent was sought from respondents, they were informed that their participation was voluntary and their refusal would not result in any negative consequences. To protect the confidentiality of the respondents, each was assigned a unique identifier which was used instead of their names. This activity was reviewed by CDC and was conducted consistent with applicable federal law and CDC policy. § §See e.g., 45 C.F.R. part 46, 21 C.F.R. part 56; 42 U.S.C. §241(d); 5 U.S.C. §552a; 44 U.S.C. §3501 et seq.

## Results

### Survivors and families interviewed

We interviewed 10 (16%) of the 63 survivors in Mubende and Kassanda districts. All survivors approached consented to participate. Of the 10 survivors interviewed, six (60%) were female. Median age was 33 years (range: 18–70 years). We further interviewed 10 members of survivor households that were not sampled for survivors; two additional household members declined to consent. Eight (80%) household members were females; all eight were mothers or spouses to the survivors and two (20%) were siblings. The median age of household members was 32 years (range: 19–57 years). Of the 10 key informants, seven (70%) were male and the median was 37 years (range: 28–58 years).

### Text coding results

Altogether, the 30 interview transcripts yielded fourteen (14) codings. **Table 1** shows the frequency of the codes used. Under the ’types of stigma’ theme, enacted stigma was the most frequently reported (24 coded segments, 55% of all codings under this theme) and institutional stigma was the least reported (eight coded segments, 18% of all codings under this theme). The ‘causes of stigma’ theme yielded 4 subthemes, with public monetary and in-kind support being the most frequently reported (26 coded segments, 34% of all codings in this theme) followed by extra attention to survivors (24 coded segments, 31% of all codings in this theme). The most frequent ‘effect of stigma’ subtheme was economic (22 coded segments, 55% of all codings in this theme), while the least frequent was social effects (six coded segments, 15% of all codings in this theme).

**Table 1:** Frequency of codes used following analysis of data on stigma among Sudan virus disease survivors and their household members in Mubende and Kassanda districts, Uganda, 2022.

Themes and sub-themes	Count of transcript segments coded
**Types of stigma**	**44**
Enacted stigma	24
Felt stigma	12
Institutional stigma	8
**Causes of stigma**	**77**
Fear that survivors who looked unwell were still ill	13
The extra attention to survivors	24
Public monetary and in-kind support	26
Risk communication about survivors was confusing	14
**Effects of stigma**	**40**
Economic effects	22
Social effects	6
Education disruption	12
**Proposed measures to control stigma**	**60**
Use of visual messages for health education and sensitization	7
Survivors should use public transport to the survivor’s clinic and have their transport refunded	18
Integrated management of interventions to avoid frequent visits	12
Private distribution of support items	23
**Total**	**219**

### Type of stigma experienced by SVD survivors and their household members, Mubende and Kassanda districts, Uganda, September 2022-January 2023

SVD survivors experienced enacted, institutional, and felt stigma.

### Enacted stigma was widely reported

All survivors interviewed, particularly those discharged during the peak of the outbreak (October 2022), experienced enacted stigma from the community in the first 2 months after discharge. There were codings related to experience of enacted stigma: [isolation and segregation by community members appeared 24 times in the 30 interviews held]. From these coded quotes, we developed the theme of “Enacted stigma based on fear from community members”. Two quotes that illustrate this theme are: *“I was treated so badly*. *People feared and isolated me*. *When I would go to the shop*, *they could not touch the money*. *[Instead] they put [out] a basket where we would drop the money and they later washed it*,*”* and *“They treated us very badly*. *The shop attendants would refuse to hold our money*, *[and] we were ignored by everyone*. *At the borehole*, *we always fetched water last because no one wanted to pump water after we had pumped*.*”*

Survivors discharged in the early stages of the epidemic reported that they were less likely to be stigmatized than those discharged in the later stages. Some believed that this was related to increased awareness about the severity of the disease by the community members over time. For example, one participant commented, *“I was welcomed well by the community and I integrated well*. *It was still early*, *and the community was not yet tired of ambulances*. *My boss offered my job back*, *but I could not do it because I was weak*.*”*

Some participants mentioned the support provided to survivors to control stigma but emphasized that survivors were still considered a threat to the community, including in their own homes. For example, one participant said, *“Survivors are still stigmatized in the communities*. *Although people cannot openly come out to talk about it…it is implied in how they relate to them*.*”* A survivor commented, “*We are still rejected*. *It will take several months because now it’s been three months*. *Maybe after six months they will be free with us*.*”* Another said, *“To date*, *some people still fear and discriminate (against) us*. *Sometimes when you come where they are*, *they don’t offer you a seat*, *so you automatically understand they still fear you*.*”*

### Institutional stigma was pronounced at schools, from teachers and students

Survivors further described experiencing institutional stigma, particularly at schools where survivor children or the child family members of affected households attended. Respondents reported that children from SVD-affected homes were sometimes denied access to school, while others were stopped from attending school by their parents due to rejection and verbal abuse from the teachers and fellow students. This experience is demonstrated in a report by one of the government officials: *“We received reports that some students from affected homesteads were denied end of term promotion exams…teachers fear to touch their books and fellow students also stigmatize them*.*”*

### Felt stigma: Survivors feared both the response of others and that they might still be infectious

Following the intense stigma from the community, some survivors felt it was best to keep to themselves. A survivor from Mubende district said *“I have not started going to the mosque*, *I fear to scare people when they see me coming*. *It’s better not to go until…the situation is better*.*”* For some survivors, the sequelae of infection made them and others worry that they might still be infectious. One participant said, *“After discharge*, *I was so happy*, *but I feared to go home because I was still weak and thought maybe the disease was still there*. *I didn’t want to go home because my body was still swollen*.*”*

### Causes of stigma among SVD survivors and their household members in Mubende and Kassanda districts, Uganda, September 2022–January 2023

#### Fear that survivors who looked unwell were still ill

Stigma among survivors was aggravated by the sequelae of infection and the fear of the disease. Sickly survivors were perceived as “still infectious”. One comment by a local government official describes this: *“Many of them still have health problems like scrotal swelling and pain*, *hearing problems*, *back pain mainly for the women*, *headache and easy irritability*. *The residual symptoms confirm the community’s myths that [SVD] cannot heal*, *further increasing the stigma among survivors*.*”* A family member also provided a related comment: *“It was hard to explain to the children why their father was still weak following discharge*, *so any sign like vomiting or coughing they would run away from him*.*”*

#### The extra attention to survivors created further stigma

Health organizations provided services to SVD survivors, including psychosocial support, in-kind household items and cash. While survivors appreciate the support, they and their household members also felt that this provided additional attention to them that aggravated both felt and enacted stigma. The frequent community visits by health organizations, who traveled to their community in branded vehicles, raised the attention of the community, as did the biweekly pickups from the community to the survivor’s clinic, which sometimes occurred in an ambulance. Two quotes that illustrate this experience are: *The use of ambulances to pick survivors for review [makes] people in the community think they are still sick*. *It is making it hard for the community to accept them as survivors*.*”* and *“Visiting their homes with so many cars*, *the neighbors wonder if somebody has become sick again*. *The frequent visits*, *frequent clinic reviews*, *the special attention with support*, *there is some level of stigma*.*”*

#### Public monetary and in-kind support for survivors caused further separation from community

Public provision of in-kind support was also reported to aggravate stigma for the survivors. As a support measure, a number of supports organizations offered cash and household items to support the survivors and their household members. It was not anticipated that this could cause any harmful effects to the beneficiaries (survivors and their household members). This was emphasized by a local government official who reported that, *“Giving them material and monetary support in public…*., *the community segregates them because they assume they have more than other members*.*”*

#### Risk communication about survivors was confusing

Community members reported that health education and community sensitization included information that was confusing to them. For example, they reported that the messaging that survivors were safe but that they also had virus in their body fluids for up to 1 year after discharge was unclear, and that it made them uncertain about how to treat survivors. One participant said, *“A lot of stigma which was promoted during health education…*. *the message that these are survivors but they still have a risk*, *they still have the virus in their semen and tears*.*”* Furthermore, the mode of sensitization was considered ineffective to some members of the community. The vehicles promoting health education through loudspeakers were not always audible or well understood. One comment from a local government official relates to the mode of communication, *“I can remember the education I received as a youth about prevention of HIV because they were showed in video form in the community squares*, *but I cannot remember all that was said a few months back when the Ebola treatment unit was opened*.*”*

### Effects of stigma on the SVD survivors and their household members in Mubende and Kassanda districts, Uganda, September 2022-January 2023

#### Economic effects

Survivors and their household members were affected in various ways due to the stigma experienced. Among the effects were economic interferences; a household member in Mubende shared that: *“The herdsman was not allowed to cross the compound to take the cows for grazing so he ran away and the cows died*,*”* while a key informant from Mubende shared: *“Most of them (the survivors) have lost their jobs*. *For the few that maintained their jobs*, *their physical health is limiting their engagement in day-to-day activities*.*”*

#### Effects on education

Additionally, stigma affected the education of school going household members to EVD survivors. Two comments by a district official and a household member describe this: *“we received reports that some students from affected homesteads were denied end of term promotion exams*, *teachers fear to touch their books and fellow students also stigmatize them*.*” “My children were discriminated by their friends; a time came when they did not want to go to school so they stayed home until examination period and so my children did not perform well this last term*.*”*

#### Social effects

Some families reportedly broke down due to the stigma. In some polygamous families where only one family was affected, the heads of family have refused to go back and denied them support. Other families lost the primary parent, resulting in child-headed homes or situations where children were sent to stay with their grandparents. For such homes, the subcounty in Mubende has tried to offer some support. A key informant in Mubende District shared that, *“Some families are now headed by children; the father died*, *the mother divorced and has refused to return in fear of getting infected”*. Furthermore, there was reportedly an increase in marital conflict caused by the close interaction among survivors. A k*ey informant in Kassanda District* shared that *“Survivors…have maintained the friendship to help console each other in case of any form of abuse*. *However*, *this close interaction among survivors has resulted in marital conflicts as wives and husbands are closer to fellow survivors than their spouses*.*”*

#### Proposed measures to control stigma among SVD survivors and their household members in Mubende and Kassanda districts, 2022

Respondents proposed a number of measures needed to control stigma. Among them was: use of visual messages for health education and sensitization in the communities and to intensify efforts to reintegrate survivors back to the community. A key informant from Kassanda District suggested: *“Intensify the integration of survivors in the community*. *We need to show that they are as normal as us and reduce on the extra attention”*. *Another key informant from Mubende District shared that; “Allow survivors use public means to the survivors clinic and have their transport refunded*, *this will reduce on the attention during pickups and drops from the clinic which causes extra attention”*. Additional measures suggested to control stigma included supporting survivors in management of their long Ebola signs and symptoms and integrated management of interventions to avoid frequent visits to survivors and their households in the community by different teams. It was suggested that all teams should move to the field together to avoid frequent visits and the so many cars that park in survivors’ compounds.

*Furthermore*, *r*espondents shared the need for additional support for improved wellbeing of survivors and their household members. These included a need for startup capital to restart their livelihoods, school fees for at least 6 months for their children while they worked to regain financial stability, and replacement of their phones, which were destroyed in the Ebola treatment unit as part of the response.

## Discussion

Survivors and household members of survivors in the 2022 SUDV outbreak in Uganda faced enacted, institutional, and felt stigma that existed despite attempts to reduce its impact. The stigma was largely caused by fear of ongoing infection. However, it was aggravated by prolonged SVD symptoms and some of the response efforts designed to support survivors.

SVD survivors and their household members faced enacted, institutional, and felt stigma, with enacted stigma being the most common. This finding is similar to those from other studies in Liberia and Sierra Leone, which found that ebola disease survivors encountered primarily enacted and perceived external stigma, rather than internalized stigma [17, 22–24]. However, a study in Sierra Leone reported higher levels of felt stigma than enacted stigma, with social isolation having the highest impact [10]. In both the Sierra Leone study and our study, enacted stigma took the form of social isolation and discrimination for both the survivors and their household members, including children [10, 25, 26]. It’s important to note that these forms of stigma are not necessarily discrete experiences and the boundaries between them can be blurred. However, it’s useful to separate them to allow for targeted responses.

The prolonged SVD symptoms that persisted for survivors after discharge were seen as exacerbating the stigmatization. Persistent health problems are well-recognized among ebola disease survivors, with the most common problems being partial loss of vision, dizziness, headache, sleeplessness, and myalgia [17, 27, 28]. In our evaluation, the community feared that the persistent symptoms represented potential ongoing contagiousness. This was compounded by the lack of clarity around risk communication messages passed on by healthcare workers, particularly around the possible ongoing presence of the virus in body fluids of survivors. It is important to provide clear messages to both survivors and their communities about the recovery process as well as information on what is and is not safe for survivors in language that is well-understood by the community. It may be beneficial to test different messages for understanding during inter-epidemic periods, so that such messaging is ready to deploy during outbreaks.

Response activities aimed at supporting survivors, including home visits from response staff, transportation in branded vehicles for survivors from the community to the survivor’s clinic, and offering of support items–particularly in public—also aggravated stigma. This finding has been observed in other settings, and scholars have suggested supporting the entire community as opposed to supporting only survivors as a way of minimizing the possibility for new discrimination [29]. In our study, some health education messages intended to sensitize the community on Ebola prevention measures further aggravated stigma. Similar findings have been obtained by various researchers; in a study of causes and consequences of ebola disease outbreak stigma among children, children perceived specific interventions initiated to contain the epidemic, such as the ‘no touch’ policy, as primary contributors to stigma [25]. Repackaging health education messages to the community for appropriateness, integrated response interventions by partners, and private distribution of support items could help control stigma.

The stigmatization of survivors affected them economically as well as socially, through loss of work. This underscores a need to support survivors with material items during the early phases of discharge before they stabilize their economic situation; support items could include clothing, food stuffs and cash [17, 30]. In Uganda, various partners did provide both monetary and material support; however, some survivors suggested that this support was not sustainable, and that provision of monitored startup capital for small businesses for sustained self-reliance would be more effective.

Members of affected households, especially children, were also stigmatized, consistent with other studies on this topic [25, 31]. Specifically, some children were denied end-of-term exams when teachers were afraid to touch their books, while in Sierra Leone, children had ropes tied around their homes preventing them from socializing and attending schools out of fear of ongoing contagion [25]. Engagement of various stakeholders, specifically in educational institutions and in the community, could help reduce stigma among children affected by ebola disease. Furthermore, supporting the children’s guardians with school fees for a few months as they regain financial stability could help ensure continuity of education for children in ebola disease -affected homesteads.

Support provided to EVD survivors to attempt to reduce the impact of stigma, which included psychosocial support and community engagements to improve acceptance, appeared to be insufficient, as stigma persisted for survivors. Similar findings have been noted in other studies, where survivors emphasized the critical need for comprehensive discharge counseling as well as facilitation of reentry into the community by professional psychosocial support counselors [17]. However, other researchers have reported better coping for EVD survivors following similar support by family, friends, and prayer groups rather than professional counselors [24, 32]. Additional studies conducted in Uganda would help contextualize the required support and approaches for application in future outbreaks.

Taken together, these results suggest a need for clearer and more effective messages to both survivors and their communities about the recovery process and safety, yet also that this should be done in a way that doesn’t escalate attention towards survivors and increase stigma. The means for achieving this could be best explored during periods in between outbreaks. Also, economic supports, both for survivor children (e.g., school fees), and adults (small business startup support) should be considered as ways of increasing social stability and an acceptance for ebola disease survivors.

### Study limitations

Because our sampling approach was based on convenience and willingness to participate, there may have been bias in who entered the study; persons who were home may have differed from those who were not. Household members who did not consent (n = 2) may have had different experiences than those who did consent (n = 10). However, this was controlled by ensuring that we attain saturation before concluding interviews for study participants.

## Conclusion

Survivors experienced mainly enacted stigma which was aggravated by the outbreak response and control activities such as additional attention to survivors from responders (including home visits by health workers, public distribution of support items, and conspicuous transport from home to the survivor’s clinic) and unclear health education messages about survivors. This affected the education of school going household members and caused economic and social disruptions. Strengthening community engagement to counteract stigma, rethinking response activities that aggravate stigma, integrated response interventions by partners, private distribution of support items, and increasing awareness and sensitization could reduce stigma among the Ebola disease survivors in future responses.
